# Developmental trajectories of metacognitive processing and executive function from childhood to older age

**DOI:** 10.1177/1747021820931096

**Published:** 2020-06-13

**Authors:** Roberto Filippi, Andrea Ceccolini, Eva Periche-Tomas, Peter Bright

**Affiliations:** 1 Institute of Education, University College London (UCL), London, UK; 2 Multilanguage and Cognition Lab, Department of Psychology and Human Development, Institute of Education, University College London (UCL), London, UK; 3 Anglia Ruskin University, Cambridge, UK; 4 Brain Research Imaging Centre, Cardiff University, Cardiff, UK

**Keywords:** Metacognitive processing, metacognition, executive function, developmental trajectories, cognitive development, bilingualism, bilingual advantage, multilingualism

## Abstract

The modern understanding of the term metacognition encompasses two levels of processing: a lower level *awareness* or *knowledge* of one’s own thoughts and a higher level *regulation* or *control* of our thinking. Metacognition, therefore, bears conceptual similarity with executive function: both are concerned with top-down monitoring and control of cognition in the service of ongoing goal-directed behaviour. Previous studies have shown a possible executive function advantage in multilingual speakers but also a possible disadvantage in metacognitive processing. To progress theory on metacognitive processing and the relationship with executive function and linguistic experience across the lifespan, we conducted a study testing 330 healthy individuals in four age groups from 7 to 80 years old. All participants performed a metacognition task and two measures of executive function, which included the Simon task and the Tower of London task. Half the participants were multilingual speakers since birth. We built developmental trajectories of metacognitive and executive function across the lifespan. The best metacognitive efficiency was observed in mid-adulthood, whereas the best executive function processing reached its peak in young adulthood. A steep cognitive decline was observed in older age, while metacognitive efficiency was preserved. Exploratory factor analysis indicated that metacognition and executive function are served by different factors across all ages. Contrary to previous findings in the bilingual literature, a multilinguistic experience conferred neither any significant advantage nor disadvantage in both executive function and metacognitive processing across the lifespan.

## Introduction

### What is metacognition

The modern understanding of metacognition as encompassing both a relatively passive (knowledge/awareness) function and an active (regulatory/control) function suggests conceptual overlap with mechanisms associated with executive function and cognitive control. In this study, we focus on the relationship between executive function and metacognitive abilities from childhood to older age.

The concept of metacognition originated in the early 1970s with an early focus on knowledge and monitoring of memory storage and retrieval, referred to as *metamemory* (Flavell, 1971). Within Flavell’s framework, metamemory skills provide optimised memory performance through the active regulation of subjective estimates of performance against actual performance (Roebers, 2017). Active *control* as well as more passive monitoring was also subsequently incorporated within a broader concept of metacognition by Flavell (1979) to describe the monitoring and control of all declarative cognitive activity. Under this framework, metacognition operates on two interacting levels: an object level (bottom-up cognitive monitoring) and a meta-level (top-down control; Nelson & Narens, 1990, 1994)). This meta-level bears similarity to Norman and Shallice’s (1986) model of executive function in which available action sequences (or schema) currently competing for selection are monitored and manipulated by a supervisory attentional system in the service of purposive, goal-directed behaviour (Fernandez-Duque et al., 2000). Arguably, therefore, the meta-level and executive systems operate comparably in the way that they modulate information via top-down control.

Intuitively, if metacognition is closely associated with mechanisms of cognitive control, we should predict that scores on tests of metacognitive ability and executive function would be highly correlated. Consistent with this view, evidence indicates that switching of attention from one task demand to another supports the ability to provide consistent/accurate performance judgements (Del Missier et al., 2010) as well as prospective confidence judgements (feeling-of-knowing) on a metamemory task involving memorising cue-target word combinations (Boduroglu et al., 2014). Successful organisation of our activities relies not just on our ability to resist strong goal-irrelevant response tendencies or to sustain attention through to the completion of a task, but also to determine the relationship between our actions and our objective performance towards a goal. Without accurate monitoring (i.e., where perceived level of performance is poorly calibrated with actual performance), we are unable to optimally regulate our knowledge or strategies in the service of goal attainment. Thus, metacognitive processing can be considered as a fundamental requirement for successful behaviour, because optimal efficiency in performance is contingent upon the calibration of actual against self-estimated progress or attainment. Consistent with this claim, for example, a large body of evidence indicates that actual achievement in educational settings is highly sensitive to calibration accuracy (for a review, see Bol & Hacker, 2012).

While it is firmly established that fluid intelligence and cognitive control are sensitive to age, with steep declines typically observed in ageing populations, the lifespan trajectory of metacognitive abilities is less certain. Some authors highlight the role of fronto-parietal networks underpinning metacognitive performance (e.g., Fleming et al., 2012; McCurdy et al., 2013), again perhaps indicating that cognitive mechanisms associated with metacognition are shared with those serving general intelligence and executive control (e.g., Barbey et al., 2012; Yoon et al., 2017). To the extent that this is true, one might predict that metacognitive skills would follow the same age-related trajectory observed for measures of executive function. Evidence for a disproportionate mismatch between confidence in abilities and actual performance on relevant tasks in older individuals compared with younger individuals is largely consistent with this prediction (e.g., Dodson et al., 2007; Hansson et al., 2008), yet other studies have indicated similar metacognitive performance in older and younger participants (e.g., Eakin et al., 2014; Halamish et al., 2011), and a recent study of perceptual and memory metacognitive ability found no evidence for a meaningful relationship between metacognition and executive function in either domain (Palmer et al., 2014).

Some research (e.g., Stankov, 1998; Stankov & Crawford, 1997) indicates that actual performance and confidence ratings differ with respect to the type of task being employed (e.g., people tend to be overconfident on tests of general knowledge and under-confident on perceptual tasks typically employed in experimental psychology). This observation has led authors (e.g., Juslin & Olsson, 1997) to claim that different tasks are associated with different (and independent) metacognitive processes. However, very high correlations observed in confidence ratings across diverse tasks, including those tapping general knowledge and perceptual discrimination (e.g., Stankov, 1998, 2000), have encouraged an alternative claim to emerge: that one metacognitive system underpins self-monitoring ability irrespective of the task undertaken (e.g., Baranski & Petrusic, 1994; Ferrell, 1995; Pallier et al., 2002; Stankov, 2000), with variations in confidence across tasks explained by general task difficulty rather than differences in the underpinning psychological processes. Moreover, the developmental literature indicates a trajectory from task specificity in metacognitive abilities in young children, with a unitary, domain general metacognitive system (i.e., one that is drawn upon irrespective of task) emerging by the age of around 15 years (e.g., Schraw et al., 1995; Veenman et al., 2004; Veenman & Spaans, 2005).

A current issue of considerable current debate is whether the limited capacity and goal-directed selectivity of our executive system can somehow be enhanced or otherwise benefit from the continuous, intense competition associated with multilingual environments (e.g., Bialystok et al., 2012; see also Paap et al., 2014, for an alternative view). Despite the large body of literature focused on this question, and the conceptual overlap between cognitive control and metacognition, very few studies have explicitly addressed the possibility that multilingualism may impact on metacognitive processing. There is evidence that bilingual university students have better insight into their reading comprehension abilities compared with their monolingual peers (Ransdell et al., 2006), that children who learned a second language in a formal context display an increased awareness and use of communicational strategies (Le Pichon Vorstman et al., 2009, 2010), and that proficient multilingualism is associated with the flexible use of grammatical (Kemp, 2009) as well as reading strategies (García et al., 1998). However, only one study has been published to date which focuses on non-linguistic metacognitive abilities in multilingual individuals. Folke and colleagues (2016) administered a computer-based two-alternative-forced-choice task. In a first-order condition, participants judged which of two simultaneously presented circles contained the most number of dots. In the second-order condition, participants stated their confidence level in each choice. In two variants of this task, bilinguals were found to respond faster than monolinguals but were significantly less metacognitively *efficient*, with efficiency mathematically determined by the difference between expected and observed performance. Thus, bilinguals were less confident in trials they completed correctly and more confident in trials where their performance was incorrect.

In the context of the purported bilingual cognitive advantage (Bialystok, 2018), evidence that there may be metacognitive *disadvantages* associated with multilingualism indicates some degree of dissociability of metacognition and executive function—and we might also observe disparity in the underlying neural signatures. In a recent review, Roebers (2017) brought together a timely review of the literature on metacognition and executive function in order to build a unifying framework for developing theoretical understanding of cognitive self-regulation. Nevertheless, to date, the literature on bilingual cognition focuses almost exclusively on executive function and neglects metacognition, possibly because the two research fields are rooted in quite different research traditions. Consolidating executive function and metacognition research and applying this to specific contexts such as multilingual cognition, therefore, constitutes an important avenue for further work.

### Rationale for this study

Studies of metacognitive processing and executive function are usually based on constrained age groups in typical, atypical, and clinical circumstances. In this study, we employ a cross-sectional design to explore how these crucial cognitive skills evolve and decline across the lifespan, from the age of 7–80 years (see Filippi et al., 2019, for a more exhaustive account of developmental approach to bilingual research). This approach has been successfully used in studies comparing the development of typical and atypical children (Annaz et al., 2009; Karmiloff-Smith et al., 2004; Thomas et al., 2009) and in a study of healthy adults (Palmer et al., 2014).

The primary objectives in the present study were to (1) broaden the focus to consider metacognition and executive function across the lifespan from childhood, through young, middle and older adulthood, (2) explore how the relationship between these abilities changes as a function of age, and (3) determine whether and how linguistic experience modulates the trajectory of these effects.

## Methods

### Participants

In all, 330 typically developing individuals took part in this study. Their age ranged from 7 to 80 years. Half of them were English monolinguals and the other half were bilinguals/multilinguals of different linguistic backgrounds. They were split into four age groups (Petry, 2002): (1) childhood 7–12 years, (2) young-adulthood, 18–35 years, (3) mid-adulthood, 36–55 years, and (4) older adulthood, 56–80 years old. Mean ages and standard deviations are reported in Table 1.

**Table 1.** Total number of participants separately by age group (in years) and linguistic group.

Age-groups	Total	*M* age	Monolinguals	*M* age	Multilinguals	*M* age
Children 7–12 years	160	9.4 (1.3)	80	9.4 (1.3)	80	9.4 (1.4)
Young adults 18–35 years	78	25.3 (4.4)	39	25.6 (4.2)	39	25.1 (4.7)
Middle-aged adults 36–55 years	42	43.9 (5.9)	21	44.5 (6.0)	21	43.3 (5.5)
Older adults 56–80 years	50	68.1 (6.0)	25	68.2 (4.7)	25	68.0 (7.1)

Standard deviations are in parentheses.

All participants completed an online questionnaire^1^ (Filippi et al., 2020) designed to establish demographic, socioeconomic, and linguistic information. Within the multilingual sample, all individuals reported acquiring two languages from birth (simultaneous bilinguals), and using them on a daily basis at home and with the extended family. A total of 59 individuals reported to be exposed to a third or a fourth language, although their level of competence in these languages was considered lower. A list of all languages is reported in the online Supplementary Material I, Table A.

All monolingual individuals reported a basic knowledge of some European languages (e.g., French, Spanish, or German) learnt at school, but were not exposed to or used a foreign language in their daily life, nor had the ability to hold a basic conversation in a language other than English.

All participants also provided socioeconomic status (SES) information indicating their highest level of education, employment, and household income. Each of the adult participants received a score depending on level of academic achievement (i.e., 1 = *no formal/primary*, 2 = *secondary*, 3 = *undergraduate*, 4 = *post-graduate*, 5 = *doctorate*). They also received a score from 1 to 4 depending on occupation (unemployed, part-time, full-time, retired), and a score from 1 to 6 depending on total household income (from less than £20,000 to more than £100,000). Scores were averaged to create a composite SES score.

### Tasks, procedure, and materials

The procedure was approved by the University Ethics Panel (FST/FREP/15/505), and was conducted in accordance with the tenets of the Declaration of Helsinki.

The experimental battery was conducted on an ASUS laptop with a mouse, standard keyboard, and a Technopro^®^ USB gamepad that was adapted with red and blue coloured stickers. All instructions were given in English.

Adult participants were tested in a quiet room made available at Anglia Ruskin University in Cambridge and at UCL–Institute of Education in London. Child participants were tested in three primary schools, two in London and one in the Cambridge area. All children gave their verbal consent before starting the session.

Participants were all assessed on a range of background measures:

#### Non-verbal reasoning

The Raven’s Advanced Progressive Matrices Set I (Raven & Court, 1998) was administered. This test of non-verbal fluid intelligence/problem solving ability consists of 12 items of increasing complexity. Each item represents a 3 × 3 matrix containing eight different black-and-white designs that are logically related and one piece missing at the bottom-right; participants are required to indicate from eight candidate pieces which piece completes the matrix. The number of correct items was recorded. All participants completed the task within 10 min.

#### Verbal working memory: digit span forwards and backwards

The 30-digit sequences from the digit span forwards (DSF) and digit span backwards (DSB) subtest of the Wechsler Adult Intelligence Scale–Fourth Edition (WAIS-IV; Wechsler, 2008) were used as a measure of the storage, maintenance, and manipulation components of verbal working memory (Richardson et al., 2010). For presentation consistency the researcher recorded each trial and played the recording via headphones to the participant. Trials began with two-digit sequences (e.g., 1–7) that the participant verbally recalled either forwards or in reverse order (DSF and DSB, respectively). As trials progress the digit sequence gradually increased to nine- (DSF) or eight-(DSB) digits. Testing was terminated if both trials of a number sequence were recalled incorrectly. The number of correct recalls for the DSF and DSB were recorded. The task lasted approximately 7 min.

#### English receptive vocabulary: British Picture Vocabulary Scale

The British Picture Vocabulary Scale: Third edition (BPVS-III; Dunn et al., 1997) consists of 14 sets of words that each contains 12 items. Difficulty levels span from simple words understood by 2–3-year-olds (e.g., ball, Set 1) to vocabulary that is above the level of an average adult (e.g., lacrimation, Set 14). The researcher orally presented the stimulus word and the participant pointed to one of four images that he or she considered most like that word. Children started with Set 8, adults with Set 11. If two or more errors were made on the starting set, then the researcher established the base set by going back a set until no more than one error was made. Next, a ceiling set was established by presenting the participant with progressively more difficult sets until eight or more errors were made on a set. Ability scores were calculated as the highest number on the ceiling set minus the total number of errors made during the assessment. Bilingual and monolingual groups were compared on their ability scores. The task lasted approximately 6 min.

## Experimental measures

### Metacognition

The dot discrimination task was programmed and conducted on PsychoPy (Version 1.82; Peirce, 2009) and was a shortened version of the task used in Folke et al. (2016) Experiment II. Experimental trials had two phases: (1) first-order performance, in which all participants had to perform a quick perceptual decision making challenge and (2) second-order performance, in which they had to rate their confidence in that decision. Metacognitive sensitivity reflects the extent that someone’s confidence rating is predictive of their accuracy in their decision (Fleming & Lau, 2014).

The trial presentation was capped at 2 s across the sample. The computation of metacognitive efficiency (Mratio) is described in the “Results” section.

Following a training phase (described below), participants completed 10 practice experimental trials and four blocks of 25 experimental trials. For each experimental trial, participants were first presented with the perceptual decision making phase where they were required to make a quick choice as to whether the circle on the left or right contained more dots, pressing the corresponding left/right cursor keys on the keyboard. One circle always contained 50 randomly located dots and the other circle would contain either fewer or more dots. Two successive correct responses resulted in the next trial being more difficult (one less dot difference); one incorrect response resulted in the task getting easier (one more dot difference; the same one-up two-down staircase procedure used in Fleming et al., 2014). Next, the metacognitive element of the trial was presented, participants had to rate their confidence that their decision was correct on a sliding scale from *less* to *more* confident using the left/right cursor keys to move the pointer and the down cursor key to submit their response. Then a new trial proceeded immediately. The perceptual decision task was response terminated but time limited, failure to respond within 1,500 ms resulted in a screen stating “Too slow” appearing for 750 ms and then a new trial was presented. Response time for the confidence judgement was unlimited.

Before the experimental task, participants were asked to view five trials to familiarise them with the stimuli; these were white outlines of a circle on the left and right both containing different numbers of white dots against a black background; beneath each circle was a number informing participants of how many dots were in the circle. Next, participants completed a training phase, where they made the quick perceptual decision as to which circle contained the most dots and then feedback appeared underneath the selected circle for 750 ms (“correct” presented in green text or “incorrect” presented in red text, or a new screen stating “too slow” if they took longer than 1,500 ms). The training phrase calibrated a participant’s difficulty level in the experimental phase. In the first trial of the training phase, there was a 20-item dot difference; a correct decision resulted in the dot difference decreasing by four and in subsequent correct trials the difference gradually decreased to one dot difference; incorrect decisions increased the dot difference. Therefore, more difficult trials were those that had a smaller difference of dots contained in the two circles. The training phase ended after participants had switched between correct and incorrect answers eight times. All training trials were excluded from analyses.

The task lasted approximately 12 min.

### Measures of executive function

#### Inhibitory control, monitoring and updating

The Simon task (Simon & Wolf, 1963) was programmed and conducted using E-Prime (Version 2.0; Schneider et al., 2007). The task was adapted from Bialystok et al. (2004, Study 1). The stimuli consisted of 18 blue stars and 18 red stars randomly presented to the left or right side of a white screen; each colour appeared an equal number of times to the left and right. The ITI was 300, 600, or 900 ms and a fixation cross appeared for 800 ms preceding the stimuli. Participants responded to red stars by pressing the red button on the left (vice versa for blue stars). During incongruent trials, the location of the stimulus and the response button do not match (red star on the right), meaning participants need to inhibit the conflicting spatial information and focus on the colour (i.e., conflict resolution). Congruent trials (red square on the left) do not require conflict resolution, meaning participants can respond faster. The task lasted approximately 2 min.

### Planning and problem solving

The Tower of London task was administered (Shallice, 1982). The task programme and software were downloaded from open-source Psychology Experiment Building Language (Version 0.13; PEBL; http://pebl.sf.net), courtesy of Mueller & Piper, 2014). The task consisted of 12 problems. Each problem required participants to use the computer mouse to move coloured discs (red, blue, and green) from their initial position to match their target position in the fewest possible moves. Participants were instructed that only one disc could be moved at a time and also that only the disc on the top of a stack could be moved. A move counter inform them how many moves they could make and how many moves they had left, and there was a maximum space for three discs per stack in the left column, two in the middle, and one in the right. Participants were also informed that there was no time limit for each problem and they were advised to think about the problem and plan their moves before they clicked on any discs. Participants clicked on the disc that they wanted to move and then clicked in the column where they wanted to place the disc. Trials ended when participants reached the move limit and the screen displayed feedback on whether or not they had successfully completed the problem. Participants then clicked to get the next trial. The initial starting position of the discs remained the same for each trial, but the target stack altered.

The trials consisted of four easy problems requiring 2–3 moves where the strategy of moving the coloured discs to match their target location worked and required minimal planning resources (Shallice, 1982). Four trials were moderate problems requiring four moves and initial moves where a disc needed to move away from its target stack (see Figure 2, where both the red and the green disc need to move away from their target stack before they can be replaced in the correct order). Four trials were difficult 5-move problems that required planning multiple sub-goals where in addition to discs initially moving away from their target location, planning was required due to the middle and right column having restricted space. Trials were presented in a fixed order where problems gradually increased in difficulty.

#### Design

This study had both a between-subject and a mixed-design in which first the developmental trajectories of metacognitive processing and executive function were built across age groups (Research Questions 1 and 2) and, subsequently, across both age and linguistic group (Research Question 3). Ability scores were obtained for the background task: BPVS III, Raven’s and Digit span and used as covariates in all comparisons. Accuracy and response time scores were calculated as the executive function tasks. Mratio was computed for metacognitive efficiency.

*T*-tests, ANOVAs, and correlation analyses were performed using SPSS version 25 for Mac. Factor analysis was performed using the “FactorAnalyzer” package with Python (https://pypi.org/project/factor-analyzer/).

## Results

### Background measures

Independent *t*-tests showed that the age difference between the language groups (English monolinguals and multilinguals) was non-significant, *t*(124) = .12, *p* = .90. Statistically equivalent age in monolinguals and bilinguals was also confirmed within each age group (*p* = .79, *p* = .60, *p* = .50, *p* = .88 for childhood, young-adulthood, mid-adulthood, and older adulthood groups, respectively).

Age-group scores and comparisons on background tests and SES scores between monolingual and multilingual individuals are reported in Table 2. Independent *t*-tests conducted for each age group indicated that English monolinguals and multilinguals were largely comparable across the measures. However, in some cases, measures of English vocabulary knowledge (BPVS), working memory (digit span backward plus forward) and SES (averaged composite scores of parental education, household income, participant higher level of education and employment status) differed significantly. We included these measures as covariates in our initial analyses. However, we conducted further tests to ascertain whether there was a correlation with the experimental measures and whether the covariates and groups were independent. We performed Pearson’s correlation analysis including all background and experimental variables, and linear regression analysis for all age groups separately. We observed overall weak correlations with all measures and the regression showed either no correlation or different directions among groups. We concluded that the independence and homogeneity assumptions were violated and therefore decided not to include the background variables as covariates here. The results of the analyses with covariates are reported in the online Supplementary Material II, Table G.

**Table 2.** Age and linguistic groups ability scores for non-verbal reasoning (Raven’s), English vocabulary knowledge (BPVS), short-term and working memory, digit span forward and backward, and socioeconomic status.

Age group	Measure	All	Monolinguals	Multilinguals	*p*
Childhood 7–12 years	Raven’s	6.6 (2.6)	6.7 (2.6)	6.5 (2.6)	.67
	BPVS	130.9 (18.0)	132.3 (16.3)	129.1 (19.4)	.33
	Digit span forward	8.5 (1.7)	8.4 (1.5)	8.5 (1.8)	.61
	Digit span backward	5.2 (1.9)	5.4 (1.9)	5.3 (2.0)	47
	Digit span total	13.6 (3.2)	13.4 (3.0)	13.8 (3.7)	.48
	Socioeconomic status	5.3 (1.1)	5.1 (1.2)	5.5 (1.0)	**.04** ^a^
Young adulthood 18–35 years	Raven’s	9.9 (2.2)	9.8 (2.2)	10.1 (2.2)	.57
	BPVS	160.1 (6.8)	162.8 (5.9)	157.3 (6.6)	**.001**
	Digit span forward	10.8 (2.3)	10.9 (2.4)	10.8 (2.1)	.81
	Digit span backward	8.0 (2.6)	7.8 (2.7)	8.2 (2.5)	.51
	Digit span total	18.9 (4.5)	18.7 (4.7)	19.0 (4.2)	.80
	Socioeconomic status	6.9 (1.1)	6.8 (1.1)	6.10 (1.0)	.43
Mid-adulthood 36–55 years	Raven’s	9.6 (1.7)	10.1 (1.9)	9.2 (1.6)	.14
	BPVS	162.3 (6.1)	165.1 (2.1)	159.6 (7.6)	**.003**
	Digit span forward	10.9 (2.6)	12.0 (2.3)	9.9 (1.9)	**.004**
	Digit span backward	8.6 (2.6)	9.3 (2.9)	7.7 (2.2)	**.04**
	Digit span total	19.4 (4.3)	21.3 (4.4)	17.6 (4.3)	**.007**
	Socioeconomic status	6.7 (1.4)	6.2 (1.6)	7.2 (1.1)	**.03**
Older adulthood 56–80 years	Raven’s	8.6 (2.4)	9.2 (1.8)	8.0 (2.8)	.10
	BPVS	166.1 (2.3)	166.0 (2.9)	166.3 (1.7)	.64
	Digit span forward	11.3 (2.4)	11.2 (2.4)	11.5 (2.4)	.69
	Digit span backward	8.1 (2.3)	8.2 (2.1)	8.0 (2.5)	.72
	Digit span total	19.4 (4.3)	19.4 (4.2)	19.5 (4.4)	.97
	Socioeconomic status	6.0 (1.5)	5.6 (1.3)	6.6 (1.6)	**.02**

BPVS: British Picture Vocabulary Scale.

Standard deviations in parentheses. Independent *t*-tests conducted by age group compare monolinguals with monolinguals differences. Statistically significant results are reported in bold.

a Where equal variances was not assumed the corrected *p* value was used.

Note that the children’s SES index is lower than that recorded for the adults. This is due to the fact that two different questionnaires were developed for this study: one for the children (to be completed by their parents) and the other for the adults. The adult questionnaire contained two additional questions: (1) employment status, that is, unemployed, part-time, full-time and retired, and (2) highest level of education, that is, A-level, undergraduate, postgraduate, and doctorate. These questions were not applicable to children and, therefore, excluded.

### How does metacognitive processing and executive function change across the lifespan? Are any effects associated with participants’ linguistic experience?

#### Metacognition

The results of first-order performance, that is, analysis of response times (measured in seconds), accuracy (measured by percentage of correct responses), and the difficulty of the trials (measured by dot difference) across all age groups and linguistic groups are reported in the online Supplementary Material III.

#### Second-order performance: metacognitive efficiency

**Figure 1.**
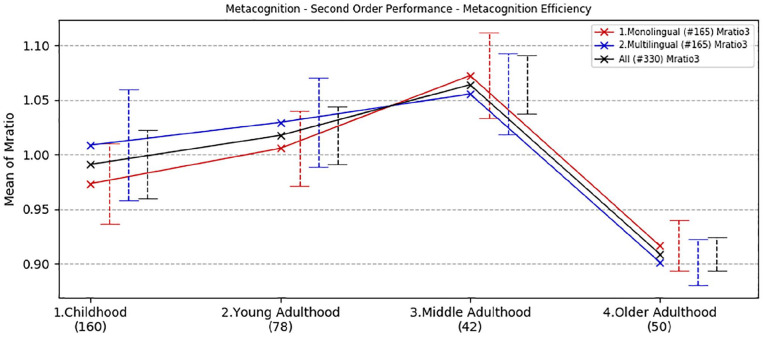
Metacognition task, second-order performance. Developmental trajectories of metacognitive efficiency (mean Mratios) with a comparison between age and language groups. Error bars show standard error.

To estimate metacognitive efficiency we used the Mratio. An Mratio was fitted to each participant’s data using a hierarchical Bayesian estimation method (see Folke et al., 2016 for a more detailed description—MATLAB code available at https://github.com/smfleming/HMM). The Mratio scores for all age and language groups are reported in Table 3 and illustrated in Figure 1.

**Table 3.** Metacognition task, second-order performance.

Age group	All participants	Monolinguals	Multilinguals
Childhood	0.99 (0.40)	0.97 (0.33)	1.01 (0.45)
Young adulthood	1.01 (0.23)	1.01 (0.21)	1.03 (0.26)
Mid-adulthood	1.06 (0.17)	1.07 (0.18)	1.05 (0.17)
Older adulthood	0.91 (0.11)	0.92 (0.12)	0.90 (0.10)

Mratio scores and standard deviations (in brackets). An Mratio of zero indicates that confidence judgements hold zero metacognitive sensitivity to the perceptual discrimination (first order) performance, with an MRatio of 1 indicating optimal metacognitive sensitivity. An MRatio value greater than 1 indicates that these participants have drawn on some other information, such as hunches (e.g., Scott et al., 2014) or knowledge of additional factors associated with task stimuli and/or performance when making their confidence judgements (Fleming, 2017; Fleming & Daw, 2017).

ANOVA showed a trend main effect of age group, *F*(3,322) = 2.13, *p* = .096, $\eta_{p}^{2}$ = .02. There was no significant effect of language group, *F*(1,322) = .031, *p* *=* .86, $\eta_{p}^{2}$ < .001, nor a significant interaction between age and language groups, *F*(3,322) = .14, *p* = .94, $\eta_{p}^{2}$ = .001.

Overall, better metacognitive performance was observed in mid-adulthood (*M* = 1.12) than all the other groups, but the differences between the groups were not statistically significant (*p* > .10).

In sum, metacognitive efficiency expressed by Mratio showed a consistent trend across all ages, with improvement through development, best performance in middle-age, and progressive decline in older age. Linguistic experience did not have any significant effect on metacognitive processing, that is, monolingual and multilingual speakers had comparable performance across all ages. Bayesian independent *t*-tests comparing metacognitive efficiency across linguistic groups in each age group indicated that the data were more than three times less likely to occur under the alternative hypothesis than the null hypothesis in all comparisons (BF^10^ < .32).

#### Executive function: inhibition and control

**Figure 2.**
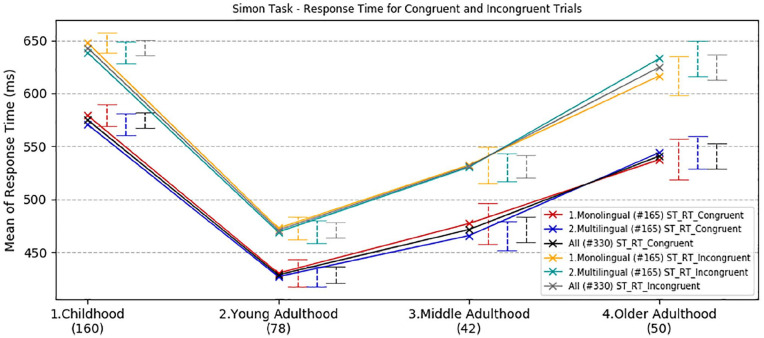
Simon task effects—developmental trajectories of mean response time in for congruent and incongruent trials, with a comparison between age and language groups. Error bars show standard error.

Response time and accuracy scores for congruent and incongruent trials in the Simon task are reported in the online Supplementary Material I, Table B and C, and illustrated in Figure 2.

For response time, a three-way ANOVA for age groups (childhood, young-adulthood, mid-adulthood and older adulthood), language groups (monolinguals, multilinguals) and congruency (congruent, incongruent) revealed a highly significant main effect of congruency overall, *F*(1,322) = 464.7, *p* < .001, $\eta_{p}^{2}$ = .59, The interaction between congruency and age group on response time was significant, *F*(3,322) = 9.0, *p* < .001, $\eta_{p}^{2}$ = .08, but not for language groups, *F*(1,322) = .58, *p* *=* .45, $\eta_{p}^{2}$ = .002. The interaction between age and language groups was also non-significant, *F*(3,322) = .22, *p* = .85, $\eta_{p}^{2}$ < .001. There was a significant overall main effect of age group, *F*(3,322) = 78.47, *p* < .001, $\eta_{p}^{2}$ = .42, but the main effect of language groups and the interaction between age and language groups were both not significant (*p* = .89).

Bonferroni adjusted pair-wise comparisons (Figure 5) showed that young adults were significantly the fastest compared with children, −159 ms, middle-aged adults −51 ms and older adults, −132 ms (*p* < .001, *p* = .005 and *p* < .001, respectively). Performance in mid-adulthood was significantly better than in childhood and in older adulthood (mean difference = −107, *p* < .001, mean difference −81 ms, *p* < .001, respectively). The older adults’ performance was comparable with children’s (mean difference = −26 ms, *p* = .26). In sum, a developmental analysis of response time in the Simon task revealed a peak in best performance with both congruent and incongruent trials in young adults. As expected, performance was worse in childhood and declined in older age. The difference in linguistic experience between individuals in all age groups did not produce any statistically significant effect (*p* = .85).

The same three-way ANOVA with accuracy scores again revealed a significant main effect of congruency, *F*(1,322) = 89.64, *p* *<* .001, $\eta_{p}^{2}$ = .22, indicating more correct responses with congruent trials, and a significant main effect of age group, *F*(3,322) = 24.64, *p* *<* .001, $\eta_{p}^{2}$ = .19. However, there was a non-significant effect of language group, *F*(1,322) = .46, *p* *=* .50, $\eta_{p}^{2}$ = .001. The interaction between congruency and age group was highly significant, *F*(3,322) = 11.41, *p* *<* .001, $\eta_{p}^{2}$ = .10. All the other interactions, that is, congruency*language group, age group*language group and congruency*age group*language group were non-significant (*p* = .73, *p* = .65, *p* = .87, respectively).

**Figure 3.**
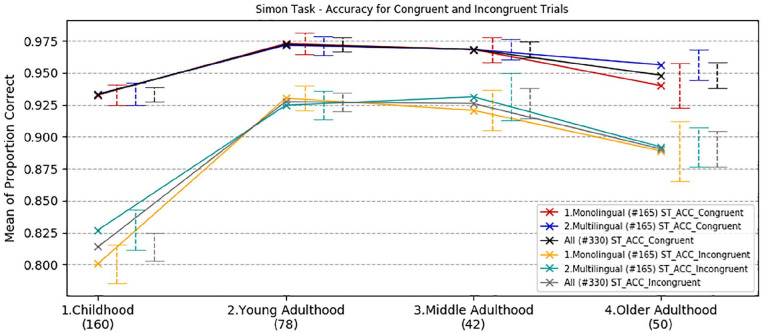
Simon task effects—developmental trajectories of mean correct responses, with a comparison between age and language groups. Error bars show standard error.

Bonferroni adjusted pair-wise comparisons (Figure 3) showed that young adults were significantly more accurate than children (mean difference = 7.6%, *p* < .001), but their performance did not differ from that of mid-adulthood and older participants (*p* = 1.0 and *p* = .13, respectively). The middle-aged adults’ and older adults’ performance compared with childhood were also significantly different (mean difference = 7.4%, *p* < .001, 4.6%, *p* = .001, respectively). A 2.8% difference in accuracy between mid-adulthood and older adulthood was not significant (*p* = .43).

As observed in the RT analysis above, the difference in linguistic experience between individuals in all age groups for accuracy in both trial conditions did not produce any statistically significant effect. Bayesian independent *t*-tests comparing Simon accuracy and RT across linguistic groups on congruent and incongruent trials conducted separately for each age group indicated that the data were more than three times less likely to occur under the alternative hypothesis than the null hypothesis in all comparisons (BF^10^ < .34).

To summarise, a developmental analysis of accuracy in the Simon task revealed a peak in best performance with both congruent and incongruent trials in young adults. Children had worse performance compared with the other groups, and the difference in linguistic experience between individuals in all age groups did not produce any significant effect.

#### Simon cost

The response time difference between congruent and incongruent trials (Simon cost) was computed for all participants across all age groups and analysed with an ANOVA. There was a highly significant main effect of age group, *F*(3,322) = 9.0, *p* *<* .001, $\eta_{p}^{2}$ = .07. There was a non-significant main effect of language group, *F*(1,322) = .58, *p* *=* .45, $\eta_{p}^{2}$ = .002, and the interaction between age and language groups was also non-significant, *F*(3,322) = .26, *p* *=* .86, $\eta_{p}^{2}$ = .002. Bayesian independent *t*-tests comparing Simon cost across linguistic groups separately for each age group indicated that the data were more than five times less likely to occur under the alternative hypothesis than the null hypothesis in children (BF^10^ = .17) and more than two times less likely in all adult groups (BF^10^ < .35 in all cases).

Bonferroni adjusted pair-wise comparisons showed that young adults had a smaller Simon cost than children, −27 ms and older adults, −41 ms (*p* = .001 and *p* < .001, respectively), but their performance was comparable with mid-adulthood (−17 ms, *p* = .32). Performance in mid-adulthood was significantly better than in childhood and in older adulthood (mean difference = −107, *p* < .001, mean difference −81 ms, *p* < .001, respectively). In sum, a developmental analysis of the Simon cost again revealed a peak in best performance in young adults. There were no significant statistical differences among the other age groups (*p* > .05 in all cases). The difference in linguistic experience between individuals in all age groups did not produce any statistically significant effect (*p* > .45 in all cases).

#### Executive function: planning

Overall accuracy, overall response time, and response time to initiate the first move on the Tower of London test were analysed by age and language group. Trials were split into two categories according to the level of complexity: (1) *moderate* (2 and 3 moves) and (2) *challenging* (4 and 5 moves). The rationale for this division is based on previous findings in bilingual research showing that multilingual speakers outperformed monolinguals only when the task presented an extra level of complexity (e.g., Filippi et al., 2012, 2015). Means and standard deviations are reported in the online Supplementary Material I, Tables D, E, and F.

The three-way ANOVA for accuracy scores revealed an overall significant main effect of trial complexity, *F*(1,322) = 271.29, *p* < .001, $\eta_{p}^{2}$ = .46. The interaction between complexity and age group was significant, *F*(3,322) = 2.75, *p* *=* .043, $\eta_{p}^{2}$ = .025.

The two-way interactions between trial complexity and language group and the three-way interaction between complexity, age group, and language group were all non-significant (*F*(1,322) = .46, *p* = .50, $\eta_{p}^{2}$ = .001, and *F*(3,322) = .27, *p* = .85, $\eta_{p}^{2}$= .002, respectively).

Tests between subjects showed a significant main effect of age group, *F*(3,322) = 35.0, *p* *<* .001, $\eta_{p}^{2}$ = .25, but the main effect of language groups and the interaction between age and language groups were both not significant (*p* = .40 and *p* = .50, respectively).

**Figure 4.**
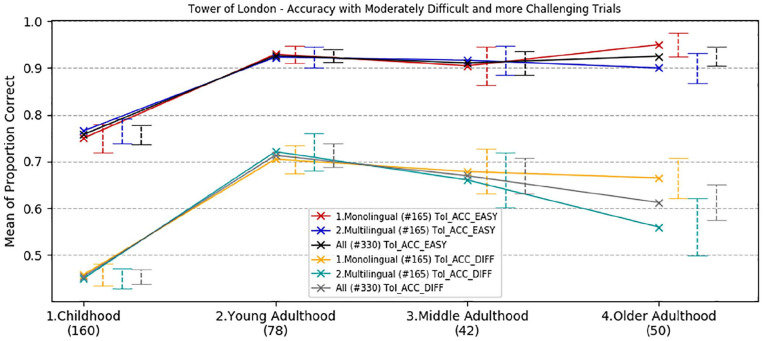
Developmental trajectories of mean correct responses in the Tower of London task, with a comparison between age and language groups. Error bars show standard error.

Bonferroni corrected pair-wise comparisons (Figure 4) showed that children were significantly less accurate than the other age group (average mean difference = difference = 18.7%, *p* < .001). All other groups had comparable performance (*p* > .60).

In sum, a developmental analysis of accuracy in the Tower of London task revealed a comparable performance in all age groups, with the exception of children who performed significantly worse than adults overall.

There was no effect of linguistic experience: monolinguals and multilinguals in all age groups had similar performance (*p* > .40). Bayesian independent *t*-tests comparing overall accuracy performance across linguistic groups in each age group indicated that the data were more than five times less likely to occur under the alternative hypothesis than the null hypothesis in the childhood group (BF^10^ < .19), more than four times less likely in the mid-adulthood (BF^10^ < .25), more than three times less likely in the mid-adulthood group (BF^10^ < .32) and more than 1.6 times less likely among the older participants (BF^10^ < .74).

ANOVA for overall mean response time to complete the test showed a significant main effect of task complexity, *F*(1,322) = 156.59, *p* *<* .001, $\eta_{p}^{2}$ = .33, and significant main effects of age group and language group, *F*(3,322) = 9.94, *p* < .001, $\eta_{p}^{2}$ = .085, *F*(1,322) = 7.05, *p* = .008, $\eta_{p}^{2}$ = .021, respectively. There was a significant interaction between complexity and age group, *F*(3,322) = 5.69, *p* = .001, $\eta_{p}^{2}$ = .050, but all other interactions were non-significant (*p* *>* .30).

**Figure 5.**
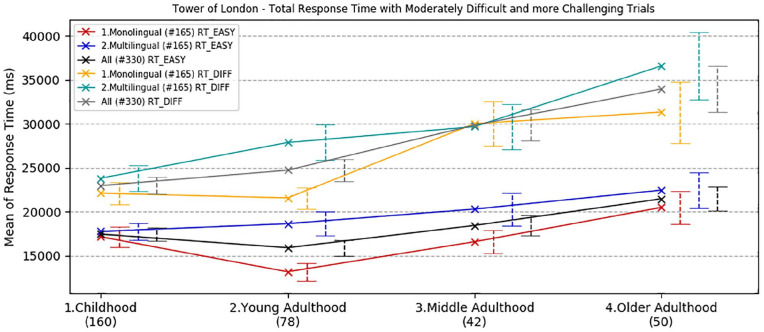
Developmental trajectories of overall mean response time for the execution of the Tower of London task (12 trials), with a comparison between age and language groups. Error bars show standard error.

Bonferroni corrected pair-wise comparisons (Figure 5) showed that older adults were significantly slower than children (mean difference = 7.5 s, *p* < .001) and young adults (mean difference = 7.3 s, *p* < .001), but their performance was comparable with the middle-aged group (*p* = .40). Monolinguals were overall 3.1 s faster than multilinguals in completing the task (*p* = .008). This difference was particularly evident and statistically significant in young adults for both moderate and challenging trials (mean difference = 5.5 s, *t*(69.8) = −3.16, *p* = .002; mean difference = 6.3 s, *t*(63.1) = −2.70, *p* = .009, respectively). Bayes factors confirmed that, in young adults, the alternative hypothesis for the linguistic group effect was over 22 times more likely under the alternative hypothesis for overall RT (BF^10^ = 22.57), a figure far lower for children (BF^10^ = .26), middle adults (BF^10^ = .31), and older adults (BF^10^ = .41).

The final ANOVA was carried out on the mean response time taken to plan the first move for both *moderate* and *challenging* trials. There was an overall significant effect of trial complexity, *F*(1,322) = 49.55, *p* < .001, $\eta_{p}^{2}$ = .133, a significant main effect of age group, *F*(3,322) = 13.01, *p* < .001, $\eta_{p}^{2}$ = .108, and language group, *F*(1,322) = 7.38, *p* = .007, $\eta_{p}^{2}$ = .022. The two-way interaction between trial complexity and age group was highly significant, *F*(3,322) = 7.67, *p* < .001, $\eta_{p}^{2}$ = .067, but all other interactions were non-significant (*p* > .20).

**Figure 6.**
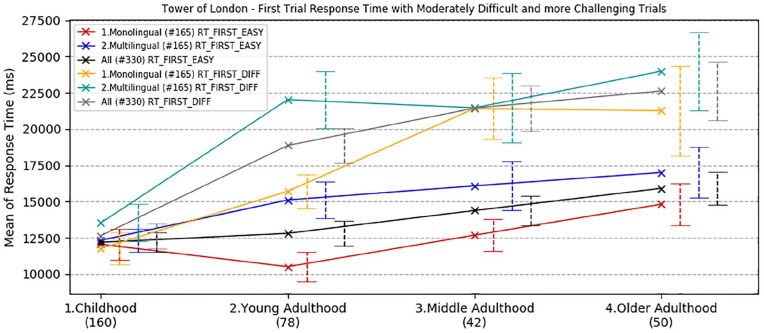
Developmental trajectories of mean response time in planning the first move in the Tower of London task, with a comparison between age and language groups. Error bars show standard error.

Bonferroni corrected pair-wise comparisons (Figure 6) showed that children were significantly faster than young adults (mean difference = 3.4 s, *p* = .010), middle-adults (mean difference = 5.5 s, *p* < .001), and older adults (mean difference = 6.8 s, *p* < .001). The other groups’ performance was comparable (*p* > .10).

Monolinguals were, on average, 2.6 s faster than multilinguals in planning the first move. Consistent with overall RT, the difference was statistically significant in young adults for both moderate and challenging trials (mean difference = 5.0 s, *t*(76) = −2.84, *p* = .006; mean difference = 6.3 s, *t*(61.5) = −2.75, *p* = .008, respectively). Bayesian analysis confirmed this considerable linguistic group effect in young adults, with the alternative hypothesis more than 20 times more likely than the null hypothesis (BF^10^ = 20.09), an effect absent in the other age groups (BF^10^ < .41 in all cases).

To summarise the overall results from the Tower of London task, it was observed that the response time for both the execution of the whole task and for planning the first move in each trial worsen with age. However, adults were more accurate: all adult age groups outperformed children in providing the right solution, irrespective of trial complexity.

### Is metacognition associated with executive function across the lifespan?

Nine variables were factor-analysed across all groups with varimax (orthogonal) rotation. The Bartlett sphericity (*p* < .001) and Kaiser–Meyer–Olkin (KMO = .781) measures verified the sampling adequacy for the analysis. The analysis yielded three factors with eigenvalues higher than 1, explaining a total of 52.65% of the variance for the entire set of variables. Table 4 shows the factor loadings after rotation.

**Table 4.** Factor analysis with varimax rotation across all groups.

	Loadings		
	Factor 1	Factor 2	Factor 3
Fluid intelligence (Raven’s)	0.666	−0.271	−0.037
Working memory (digit span backward + forward)	0.653	−0.25	0.015
Tower of London: accuracy Moderate trials	0.511	−0.064	−0.027
Tower of London: accuracy Challenging trials	0.671	−0.128	0.034
Simon task: accuracy congruent trials	0.096	−0.304	0.057
Simon task: accuracy incongruent trials	0.353	−0.154	−0.031
Simon task: response time congruent trials	−0.217	0.958	0.039
Simon task: response time incongruent trials	−0.341	0.814	0.047
Metacognition (Mratio)	−0.008	0.037	0.997
Eigenvalues	3.71	1.20	1.04
Percent of total variance	20.87%	20.62%	11.16%
Cumulative variance	52.65%

Factor 1 showed higher loadings towards more difficult tasks, that is, challenging trials in the Tower of London and, to a lesser extent, incongruent trials in the Simon task. Performance on digit span and Raven’s matrices also loaded highly, indicative of a shared latent executive function/fluid intelligence factor underpinning performance on these tasks. This first factor explained 20.87% of the variance.
Factor 2 was mainly represented by the Simon task with congruent and incongruent trial response time. This factor may represent both sustained attention to the task and inhibitory control or conflict monitoring. Factor 2 explained 20.65% of the variance. The third factor was uniquely represented by the metacognition task explaining 11.16% of the variance.

**Figure 7.**
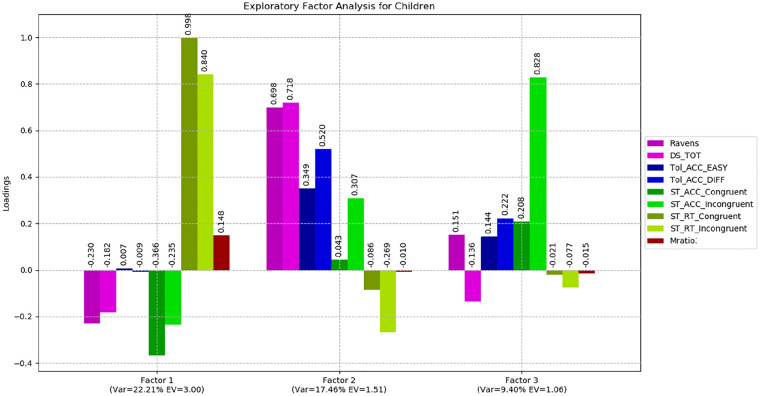
Exploratory factor analysis for children.

**Figure 8.**
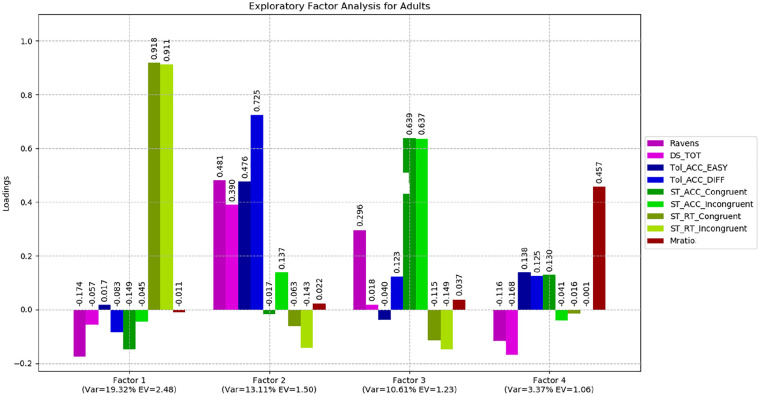
Exploratory factor analysis for adults.

Two separate exploratory factor analyses were carried out for children and for adults. The results were largely consistent with our full sample findings (see Figures 7 and 8). Rotated matrices are reported in the online Supplementary Material IV.

Overall, factor analysis has shown that metacognitive processing does not appear to recruit the same mechanism associated with performance on the tests of working memory, fluid intelligence and executive function.

## Discussion

Our primary objective in this study was to chart the developmental trajectories of performance on measures of metacognitive processing and executive function across the life span. For this purpose, a large sample of healthy individuals (*N* = 330) from 7 to 80 years old were tested on the same tasks measuring executive function (inhibitory control, conflict monitoring, and updating and strategic planning), working memory, fluid intelligence, and metacognition. A second objective was to identify the relationship between metacognition and executive function and consider how this relationship changes across the lifespan. Finally, to address the viability of the bilingual cognitive advantage hypothesis, we determined whether the trajectory of these effects is modulated by participants’ linguistic experience.

### Developmental trajectories of metacognitive processing

We administered a two-alternative-forced-choice task in which participants attempted to identify which one of two circles presented on screen contained more dots (within a 2-s response window) and subsequently rate their level of confidence in their choice. Metacognitive efficiency was computed and expressed by Mratio (Barrett et al., 2013; Fleming & Lau, 2014) and compared across the four age groups. The developmental trajectory showed that participants in the mid-adulthood group (36–55 years old) demonstrated best metacognitive efficiency, that is, they tended to feel more confident in trials they completed correctly and less confident in trials where their performance was not correct. The childhood group (7–12 years old) showed overall worst metacognitive performance. A steep metacognitive efficiency decline was observed in older age (56–80 years old).

Participants’ linguistic experience did not produce any significant effect: both monolingual and multilingual speakers’ trajectories were comparable overall.

This result is inconsistent with a study by Folke et al. (2016), which employed the same task but reported a metacognitive disadvantage in multilingual young adults in comparison to monolingual peers. Beyond the more constrained age range, the most evident difference between that study and the present one is that, of the 31 bilinguals, just over half did not begin learning a second language until after the age of 6. In our present study which included a group of 78 young adults, all 165 multilingual participants were simultaneous bilinguals, exposed to two or more languages from birth. Metacognitive processing in bilingualism is a new area of research, and it is therefore not possible to draw firm conclusions regarding the relevance of bilingualism to the development of metacognitive efficiency. Nevertheless, in supporting either a disadvantage or no advantage at all, these studies together (which, to our knowledge, are the only studies to date focusing on metacognition in bilingual research using this method) are most consistent with the position that bilingualism does not confer benefit in this regard: there is no general metacognitive bilingual advantage.

### Developmental trajectories of executive function and planning

The Simon task was used to measure executive function across the lifespan. Consistent with previous work (for a review see Van der Lubbe & Verleger, 2002), the developmental trajectory showed the best reaction time performance in young adults when compared with the other age groups. Older adults showed a significant decline both in terms of response time and accuracy, especially on incongruent trials. This result is in line with previous research showing a progressive improvement of inhibitory control and monitoring in childhood and young adulthood, and a decline associated with ageing (e.g., De Luca et al., 2003; De Luca & Leventer, 2010; Hämmerer et al., 2010; Lorsbach & Reimer, 2008; Rabbitt et al., 2001). Nevertheless, contrary to previous psycholinguistic research (e.g., Bialystok et al., 2004), there was no significant effect of multilingualism across the lifespan. The development and decline of inhibitory control and monitoring followed the same trajectory in both monolingual and multilingual speakers. However, although the Simon test is widely employed as a measure of inhibition, we also acknowledge that reported correlations of performance across tests designed to measure inhibition are frequently low, and that this observation has led authors to question the convergent validity of the term, and therefore its usefulness in the literature (e.g., Paap et al., 2020; Rey-Mermet et al., 2018).

On the Tower of London task, designed to assess strategic executive function and planning, all groups showed comparable accuracy performance (trials successfully completed) when the demand of the task was less challenging, that is, with trials requiring fewer moves to completion. However, the trajectory was different when the trials placed greater demands on strategic planning. Young adults had best performance and a progressive decline was observed with ageing, especially in the multilingual population, although the difference was not statistically significant. Developmental trajectories of response time revealed a different pattern, in which monolingual speakers in general, and especially in young adulthood, showed significantly faster overall performance in completing the task than multilingual peers in all age groups. This difference was particularly significant when the time to perform the first move was considered. English monolingual young adults demonstrated a faster response time with planning than multilingual peers both for less demanding and more challenging trials. These findings are consistent with a study of 45 young adults by Naeem et al. (2018), in which monolinguals were also found to perform better on the Tower of London test, once SES of participants was taken into account. Another recent study by Papageorgiou et al. (2019) in older participants showed statistically equivalent performance in monolinguals and bilinguals, with a trend towards a bilingual disadvantage in response times on this task. Together, these findings clearly do not support the existence of a genuine cognitive advantage in executive function which is underpinned by multilanguage acquisition. To the extent that there is a bilingual advantage, it appears not to extend to planning and sustained cognitive control of behaviour towards a goal.

### Links between metacognition and executive function

Exploratory factor analysis is a statistical method designed to identify latent factors or constructs that contribute to performance across multiple variables entered into the model. The results presented here clearly indicate that metacognitive efficiency (as measured by Mratio) is independent of the mechanism(s) driving performance on our other tasks. We identified strong correlations between Raven’s matrices, digit span and Tower of London performance, and Factor 1, indicating that a working memory/executive attention construct underpins performance on these tests. Response times for both congruent and incongruent trials on the Simon test loaded strongly on a second factor. The key finding, however, was that our measure of metacognition showed a negligible correlation with Factors 1 and 2 and instead independently loaded on the third extracted factor in our full sample. Over 99% of the variance in our metacognition variable was unique, indicating that virtually zero variance was shared with the other variables in our model. This finding is consistent with studies of confidence judgements on fluid intelligence tasks (e.g., Stankov, 2000) metacognitive efficiency in the domains of memory and perception (Palmer et al., 2014), but not with studies employing “feeling of knowing” (Souchay & Isingrini, 2004), raising the possibility that there may be different forms of metacognitive processing of which only some share the same cognitive mechanisms underpinning executive function. Nevertheless, on the basis of the present findings we conclude that perceptual metacognitive efficiency relies on mechanisms distinct from those serving working memory and executive planning abilities. As outlined in our “Introduction” we suggest that the balance of evidence in the literature is most consistent with there being a domain general metacognitive ability in older children and adults, which is drawn upon irrespective of task characteristics, and we therefore hypothesise that cognitive mechanisms underpinning metacognitive skills may be quite independent from those serving executive function/cognitive control beyond the domain of perception-based discrimination performance. However, further research, undertaken with a diverse range of metacognitive tasks, is required to formally address this question. We also encourage efforts to address the developmental trajectories of these cognitive systems in children.

### The effects of multilingualism on metacognition and executive function across the lifespan

Across the tests presented in the current study, comparable levels of performance were observed in monolingual and bilingual groups, and this finding applied in all age groups (although it should be noted that numbers of participants in our older age groups were comparatively small). The only significant effect favoured monolingual participants, who performed the Tower of London task faster than multilinguals (particularly the case in the younger age groups). However, the level of accuracy was comparable in both linguistic groups and across all ages. The evidence base for the bilingual cognitive advantage (Bialystok, 2018) has been robustly challenged in the recent literature on bilingualism (e.g., de Bruin et al., 2015; Goldsmith & Morton, 2018; Paap et al., 2015) and the present findings are also incompatible with the primary claim of this theory: that the process of becoming bilingual/multilingual confers domain general benefits in executive function and cognitive control. Our present findings, based on a considerably larger sample than that typically employed in bilingualism research, provide further confirmatory evidence not only that there is no statistically meaningful advantage for bilinguals on widely established tests of executive function, but also that bilingualism does not appear to offer advantages in metacognitive efficiency.

## Conclusion

Our findings indicate that metacognitive efficiency and accuracy on measures of executive function show similar, non-linear trajectories across the lifespan, with children performing disproportionately worse than young and middle-aged adults, and older adults showing a marked decline. However, despite these trends, there was no statistical evidence for a relationship between metacognition and our sampled components of executive function (strategic planning, fluid intelligence, conflict monitoring and working memory) indicating that these broad cognitive abilities may be served by independent cognitive mechanisms. Our findings, based on carefully matched groups of participants, also indicate that bilingualism does not appear to confer advantages either in executive function or metacognition in children (over the age of 6) or adults of any age.

## Supplemental Material

QJE-STD-20-136.R2-Supplementary_Material – Supplemental material for Developmental trajectories of metacognitive processing and executive function from childhood to older age Click here for additional data file. Supplemental material, QJE-STD-20-136.R2-Supplementary_Material for Developmental trajectories of metacognitive processing and executive function from childhood to older age by Roberto Filippi, Andrea Ceccolini, Eva Periche-Tomas and Peter Bright in Quarterly Journal of Experimental Psychology

## Notes

1. Children questionnaire data were provided by their parents.
