# Supplementary material for: Developmental trajectories of metacognitive processing and executive function from childhood to older age
Source: Q J Exp Psychol (Hove). 2020 Jun 13;73(11):1757–73. doi: 10.1177/1747021820931096 (PMC7715991; doi:10.1177/1747021820931096)
Supplement: QJE-STD-20-136.R2-Supplementary_Material – Supplemental material for Developmental trajectories of metacognitive processing and executive function from childhood to older age [file QJE-STD-20-136.R2-Supplementary_Material.docx]

Supplementary Material for

**Developmental trajectories of metacognitive processing and executive function from childhood to older age**

Roberto Filippi, Andrea Ceccolini, Eva Periche-Tomas, Peter Bright

**Supplementary Material I**

**Table A**: Linguistic backgrounds of multilinguals participants. They were all fluent in English plus the languages listed in the table.

|  |  |  |
| --- | --- | --- |
| **Age Group** | **Languages** | **No.** |
| **Childhood**  (7-12 years old) | Cantonese | 1 |
|  | Czech | 1 |
|  | Dutch | 3 |
|  | Finnish | 1 |
|  | French | 12 |
|  | French/Italian | 1 |
|  | Greek | 1 |
|  | Hebrew | 1 |
|  | Italian | 4 |
|  | Latvian | 1 |
|  | Polish | 7 |
|  | Portuguese | 2 |
|  | Arabic | 1 |
|  | Russian | 7 |
|  | Somali | 1 |
|  | Spanish | 4 |
|  | Swedish | 2 |
|  | Tamil | 2 |
|  | Turkish | 3 |
|  | Urdu | 1 |
|  | Vietnamese | 1 |
|  | Albanian/Russian | 2 |
|  | Bulgarian/Macedonian | 1 |
|  | French/Norwegian | 1 |
|  | Greek/French | 1 |
|  | Italian/French | 1 |
|  | Italian/Finnish | 2 |
|  | Italian/Spanish | 1 |
|  | Korean/Japanese | 1 |
|  | Portuguese/French | 1 |
|  | Portuguese/Italian | 2 |
|  | Russian/French | 1 |
|  | Spanish/French | 2 |
|  | Spanish/Portuguese | 1 |
|  | Spanish/Swedish | 1 |
|  | Spanish/Basque | 1 |
|  | German/French | 1 |
|  | Urdu/Pashto | 1 |
|  | Russian/Italian/French | 2 |
|  |  |  |
| **Young Adulthood**  (18-35 Years Old) | Hindi | 1 |
|  | Punjabi | 1 |
|  | Tagalog | 1 |
|  | Gujarati | 2 |
|  | Twi | 1 |
|  | Russian | 1 |
|  | Cantonese | 3 |
|  | Jamaican Patwa | 1 |
|  | Greek | 2 |
|  | Dhivehi | 1 |
|  | Arabic | 1 |
|  | Irish | 1 |
|  | Italian | 3 |
|  | Albanian/Italian | 1 |
|  | Belarusian/Russian | 1 |
|  | Catalan/Spanish | 1 |
|  | Portuguese/Spanish | 1 |
|  | French/Spanish | 1 |
|  | French/Dutch | 1 |
|  | Cantonese/Mandarin | 2 |
|  | Spanish/French | 1 |
|  | Chinese/Malay | 2 |
|  | Punjabi/Hindu | 2 |
|  | Turkish/Slavic | 1 |
|  | Romanian/French | 1 |
|  | French/German | 1 |
|  | German/Cantonese | 1 |
|  | German/Polish | 1 |
|  | Malayalan/Hindu | 1 |
|  | Romanian/Italian | 1 |
|  |  |  |
| **Middle Adulthood**  (36-55 Years Old) | Jamaican Creole | 1 |
|  | Mandarin | 2 |
|  | Danish | 1 |
|  | Dutch | 1 |
|  | Greek | 2 |
|  | Italian | 1 |
|  | Portuguese | 2 |
|  | Russian | 2 |
|  | Malay | 1 |
|  | Hebrew | 1 |
|  | Bengali | 1 |
|  | French | 1 |
|  | Basque/Spanish | 1 |
|  | French/Italian | 1 |
|  | Italian/Hebrew | 1 |
|  | Spanish/Catalan | 1 |
|  | Armenian/Russian | 1 |
|  |  |  |
| **Older Adulthood**  (56-80 Years Old) | Arabic | 1 |
|  | French | 8 |
|  | Italian | 2 |
|  | Norwegian | 1 |
|  | Polish | 1 |
|  | Spanish | 1 |
|  | Dutch/French | 1 |
|  | French/German | 1 |
|  | French/Spanish | 4 |
|  | French/Portuguese | 1 |
|  | French/Dutch | 1 |
|  | French/Italian | 1 |
|  | Italian/Spanish | 1 |
|  | Spanish/French | 1 |

Table B: Mean reaction times (milliseconds) and standard deviations (in brackets) in the Simon Task.

|  | **ALL** | | **Monolinguals** | | **Multilinguals** | |
| --- | --- | --- | --- | --- | --- | --- |
|  | Congruent | Incongruent | Congruent | Incongruent | Congruent | Incongruent |
| Childhood | 575 (90.4) | 643 (89.4) | 579 (89.6) | 647 (85.5) | 571 (91.4) | 638 (93.6) |
| Young Adulthood | 429 (68.9) | 471 (65.5) | 430 (79.1) | 473 (66.2) | 427 (57.9) | 469 (65.6) |
| Middle Adulthood | 472 (76.2) | 531 (69.4) | 477 (88.5) | 532 (78.6) | 466 (63.3) | 530 (60.6) |
| Older Adulthood | 541 (85.6) | 624 (86.9) | 538 (96.1) | 617 (91.1) | 544 (75.6) | 633 (83.5) |

Table C: Mean accuracy (percent correct) and standard deviations (in brackets) in the Simon Task.

|  | **ALL** | | **Monolinguals** | | **Multilinguals** | |
| --- | --- | --- | --- | --- | --- | --- |
|  | Congruent | Incongruent | Congruent | Incongruent | Congruent | Incongruent |
| Childhood | 0.93 (0.07) | 0.81 (0.14) | 0.93 (0.07) | 0.80 (0.14) | 0.93 (0.08) | 0.83 (0. 14) |
| Young Adulthood | 0.97 (0.05) | 0.93 (0.06) | 0.97 (0.05) | 0.93 (0.06) | 0.97 (0.05) | 0.92 (0.06) |
| Middle Adulthood | 0.97 (0.04) | 0.93 (0.08) | 0.97 (0.05) | 0.92 (0.07) | 0.97 (0.04) | 0.93 (0.08) |
| Older Adulthood | 0.95 (0.07) | 0.89 (0.10) | 0.94 (0.09) | 0.89 (0.12) | 0.96 (0.07) | 0.89 (0.07) |

**Table D**: Mean accuracy (percent correct) and standard deviations (in brackets) in the Tower of London task with comparison between moderate and challenging trials.

|  | **ALL** | | **Monolinguals** | | **Multilinguals** | |
| --- | --- | --- | --- | --- | --- | --- |
|  | Moderate | Challenging | Moderate | Challenging | Moderate | Challenging |
| Childhood | 0.76 (0.26) | 0.46 (0.20) | 0.75 (0.27) | 0.46 (0.21) | 0.77 (0.24) | 0.45 (0.19) |
| Young Adulthood | 0.93 (0.13) | 0.72 (0.25) | 0.93 (0.11) | 0.71 (0.19) | 0.92 (0.14) | 0.72 (0.25) |
| Middle Adulthood | 0.91 (0.16) | 0.67 (0.24) | 0.90 (0.19) | 0.68 (0.22) | 0.92 (0.14) | 0.66 (0.27) |
| Older Adulthood | 0.93 (0.15) | 0.62 (0.27) | 0.95 (0.13) | 0.67 (0.21) | 0.90 (0.16) | 0.56 (0.31) |

**Table E**: Mean overall response time (milliseconds) and standard deviations (in brackets) in the Tower of London task with comparison between moderate and challenging trials.

|  | **ALL** | | **Monolinguals** | | **Multilinguals** | |
| --- | --- | --- | --- | --- | --- | --- |
|  | Moderate | Challenging | Moderate | Challenging | Moderate | Challenging |
| Childhood | 17470 (9630) | 22976 (12137) | 17170 (10414) | 22128 (11552) | 17770 (8832) | 23824 (12712) |
| Young Adulthood | 15926 (8084) | 24734 (10763) | 13190 (6417) | 21582 (7667) | 18663 (8711) | 27887 (12473) |
| Middle Adulthood | 18462 (7552) | 29858 (11567) | 16608 (6118) | 30028 (11588) | 20317 (8500) | 29689 (11829) |
| Older Adulthood | 21489 (9686) | 33969 (18336) | 20509 (9226) | 31343 (17429) | 22469 (10219) | 36595(19191) |

**Table F**: Mean response time for planning the first trial (milliseconds) and standard deviations (in brackets) in the Tower of London task with comparison between moderate and challenging trials.

|  | **ALL** | | **Monolinguals** | | **Multilinguals** | |
| --- | --- | --- | --- | --- | --- | --- |
|  | Moderate | Challenging | Moderate | Challenging | Moderate | Challenging |
| Childhood | 12196 (8479) | 12650 (10760) | 12059 (9697) | 11770 (9882) | 12332 (7115) | 13529 (11568) |
| Young Adulthood | 12814 (7458) | 18869 (10553) | 10515 (6315) | 15708 (7258) | 15114 (7873) | 22030 (12347) |
| Middle Adulthood | 14386 (6684) | 21441 (10268) | 12686 (5007) | 21414 (9710) | 16087 (7774) | 21467 (11038) |
| Older Adulthood | 15914 (8041) | 22629 (14411) | 14820 (7173) | 21272 (15449) | 17009 (8836) | 23986 (13472) |

**Supplementary Material II**

Table G: Pearson's correlation analyses

| **Experimental Variables** | **Background Variables** | **Correlation** | **t** | **Sig.(2-tailed)** |
| --- | --- | --- | --- | --- |
| **Metacognition**  (Second Order Performance) | Ravens | -0.050 | -0.902 | 0.368 |
|  | BPVS | -0.108 | -1.970 | 0.050 ** |
|  | Digit Span | 0.002 | 0.045 | 0.964 |
|  | SES | 0.008 | 0.140 | 0.889 |
| **Tower of London**  (Accuracy Easy Trials) | Ravens | 0.363 | 7.048 | 0.000 ** |
|  | BPVS | 0.389 | 7.651 | 0.000 ** |
|  | Digit Span | 0.352 | 6.801 | 0.000 ** |
|  | SES | 0.210 | 3.898 | 0.000 ** |
| **Tower of London**  (Accuracy Difficult Trials) | Ravens | 0.517 | 10.927 | 0.000 ** |
|  | BPVS | 0.419 | 8.357 | 0.000 ** |
|  | Digit Span | 0.463 | 9.448 | 0.000 ** |
|  | SES | 0.231 | 4.298 | 0.000 ** |
| **Tower of London**  (RT Easy Trials) | Ravens | -0.100 | -1.826 | 0.069 |
|  | BPVS | -0.025 | -0.448 | 0.654 |
|  | Digit Span | -0.030 | -0.542 | 0.588 |
|  | SES | 0.047 | 0.857 | 0.392 |
| **Tower of London**  (RT Difficult Trials) | Ravens | 0.126 | 2.302 | 0.022 ** |
|  | BPVS | 0.202 | 3.743 | 0.000 ** |
|  | Digit Span | 0.233 | 4.332 | 0.000 ** |
|  | SES | 0.174 | 3.202 | 0.001 ** |
| **Tower of London**  (RT First Move Easy Trials) | Ravens | 0.016 | 0.287 | 0.774 |
|  | BPVS | 0.066 | 1.194 | 0.233 |
|  | Digit Span | 0.056 | 1.012 | 0.312 |
|  | SES | 0.116 | 2.107 | 0.036 ** |
| **Tower of London**  (RT First Move Difficult Trials) | Ravens | 0.305 | 5.790 | 0.000 ** |
|  | BPVS | 0.336 | 6.456 | 0.000 ** |
|  | Digit Span | 0.363 | 7.058 | 0.000 ** |
|  | SES | 0.271 | 5.090 | 0.000 ** |
| **Simon Task**  (RT Congruent Trials) | Ravens | -0.436 | -8.778 | 0.000 ** |
|  | BPVS | -0.482 | -9.968 | 0.000 ** |
|  | Digit Span | -0.396 | -7.803 | 0.000 ** |
|  | SES | -0.335 | -6.436 | 0.000 ** |
| **Simon Task**  (RT Incongruent Trials) | Ravens | -0.507 | -10.659 | 0.000 ** |
|  | BPVS | -0.510 | -10.733 | 0.000 ** |
|  | Digit Span | -0.427 | -8.543 | 0.000 ** |
|  | SES | -0.360 | -6.999 | 0.000 ** |
| **Simon Task**  (Accuracy Congruent Trials) | Ravens | 0.258 | 4.836 | 0.000 ** |
|  | BPVS | 0.219 | 4.070 | 0.000 ** |
|  | Digit Span | 0.166 | 3.048 | 0.002 ** |
|  | SES | 0.127 | 2.322 | 0.021 ** |
| **Simon Task**  (Accuracy Inongruent Trials) | Ravens | 0.482 | 9.965 | 0.000 ** |
|  | BPVS | 0.421 | 8.406 | 0.000 ** |
|  | Digit Span | 0.315 | 6.016 | 0.000 ** |
|  | SES | 0.166 | 3.055 | 0.002 ** |

**Supplementary Material III**

Metacognition: first order analyses

Two-way between-subjects ANOVAs for age group and language group for mean response time showed a highly significant main effect of age group, *F*(3,322)=31.97, *p*<.001, η_p_^2^=.23. There was no significant effect of language group, *F*(1,322)= 1.94, *p=*.16, η_p_^2^=.005, nor a significant interaction between age and language groups, *F*(3,322)=1.38, *p*=.25, η_p_^2^=.013.

Bonferroni corrected pair-wise comparisons, showed and that older adults were significantly slower when compared to all other age groups (average mean difference = 138 ms, *p*<.001). In summary, despite the fact that the trial presentation was capped to 2 seconds, an effect of age on response time was observed with older participants being slower than all other groups. The effect of age was comparable between monolingual and multilingual participants. However, multilingual older adults showed slower performance than monolingual peers (mean difference = 58 milliseconds), and this difference was statistically significant, *t*(48)=-2.39, *p*=.021.

The same analysis was repeated for accuracy. ANOVA again revealed a significant main effect of age group, *F*(3,322)=14.71, *p*<.001, η_p_^2^=.12 and a non-significant effect of language group, *F*(1,322)=.94, *p=*.33, η_p_^2^<.003. There was a strong trend in the interaction between age and language groups, *F*(3,322)=2.61, *p*=.051, η_p_^2^=.024.

Bonferroni corrected pair-wise comparisons showed that the older adults' performance was 5% less accurate when compared to young adults (*p*<.001) and 4% in comparison with middle-aged adults (*p*=.004)^^[[1]](#footnote-1)^^.

The children's performance was 4% less accurate than young adults (*p*<.001)^^[[2]](#footnote-2)^^.

All the other age-group comparisons were non-significant. Multilingual children and older adults had different trajectories when compared to monolinguals, with a steeper performance decline among multilingual adults as a function of age. Multilingual children were more accurate and older multilingual participants were less accurate than their monolingual peers, but the difference was statistically non-significant in both cases (mean difference = 2%, *t*(158)=-1.55, *p*=.12; mean difference = 3%, *t*(48)=1.62, *p*=.11, respectively).

We also applied an ANOVA to establish whether there were significant differences between age and language groups with regard to trial difficulty (defined by the difference in the number of dots contained in the two simultaneously presented circles, where a larger difference makes the trial easier and a smaller difference makes the trial more difficult, as outlined in the Methods section).

There was a main significant effect of age group, *F*(3,322)= 23.73, *p*<.001, η_p_^2^=.18 and a non-significant effect of language group, *F*(1,322)=3.31, *p=*.07, η_p_^2^=.010. The interaction between age and language groups was also non-significant, *F*(3,322)=.71, *p*=.55, η_p_^2^=.007. Bonferroni corrected pair-wise comparisons showed that the children's performance was significantly worse than all other age groups (average mean difference = 2.4, *p*<.001).

**Supplementary Material IV**

Table H: Factor analysis with varimax rotation for children.

|  |  | **Loadings** |  |
| --- | --- | --- | --- |
|  | **Factor 1** | **Factor 2** | **Factor 3** |
| Fluid Intelligence (Ravens) | -0.23 | 0.698 | 0.151 |
| Working Memory (digit span backward+forward) | -0.182 | 0.718 | -0.136 |
| Tower of London: Accuracy  Moderate Trials | 0.007 | 0.349 | 0.144 |
| Tower of London: Accuracy  Challenging Trials | -0.009 | 0.52 | 0.222 |
| Simon task: Accuracy congruent Trials | -0.366 | 0.043 | 0.208 |
| Simon task: Accuracy incongruent Trials | -0.235 | 0.307 | 0.828 |
| Simon task: Response Time Congruent Trials | 0.998 | -0.086 | -0.021 |
| Simon task: Response Time Incongruent Trials | 0.84 | -0.269 | -0.077 |
| Metacognition (Mratio) | 0.148 | -0.01 | -0.015 |
|  |  |  |  |
| *Eigenvalues* | 3.00 | 1.51 | 1.06 |
| *Percent of Total Variance* | 22.20% | 17.50% | 9.40% |
| *Cumulative Variance* | 49.10% | | |

Table I: Factor analysis with varimax rotation for adults.

|  |  | **Loadings** |  |  |
| --- | --- | --- | --- | --- |
|  | **Factor 1** | **Factor 2** | **Factor 3** | **Factor 4** |
| Fluid Intelligence (Ravens) | -0.174 | 0.481 | 0.296 | -0.116 |
| Working Memory (digit span backward+forward) | -0.057 | 0.39 | 0.018 | -0.168 |
| Tower of London: Accuracy  Moderate Trials | 0.017 | 0.477 | -0.04 | 0.138 |
| Tower of London: Accuracy  Challenging Trials | -0.083 | 0.725 | 0.123 | 0.125 |
| Simon task: Accuracy congruent Trials | -0.149 | -0.017 | 0.639 | 0.129 |
| Simon task: Accuracy incongruent Trials | -0.045 | 0.137 | 0.637 | -0.041 |
| Simon task: Response Time Congruent Trials | 0.918 | -0.063 | -0.115 | -0.016 |
| Simon task: Response Time Incongruent Trials | 0.911 | -0.143 | -0.149 | 0 |
| Metacognition (Mratio) | -0.011 | 0.022 | 0.037 | 0.457 |
|  |  |  |  |  |
| *Eigenvalues* | 2.48 | 1.5 | 1.23 | 1.06 |
| *Percent of Total Variance* | 19.30% | 13.10% | 10.60% | 3.40% |
| *Cumulative Variance* | 46.40% | | |  |

1. [↑](#footnote-ref-1)
2. This result, at odds with the staircase procedure, is likely due to a smaller sample size in the older population. However, this difference has not affected the second order performance, where metacognitive efficiency was non-significant across all age groups. [↑](#footnote-ref-2)
